# The Primary Care Medical Record Industry in Canada and Its Data Collection and Commercialization Practices

**DOI:** 10.1001/jamanetworkopen.2025.7688

**Published:** 2025-05-05

**Authors:** Sheryl Spithoff, Leslie Vesely, Brenda McPhail, Robyn K. Rowe, Lana Mogic, Quinn Grundy

**Affiliations:** 1 Department of Family and Community Medicine, University of Toronto, Toronto, Ontario, Canada; 2 Women’s College Hospital, Toronto, Ontario, Canada; 3 Faculty of Social Sciences, McMaster University, Hamilton, Ontario, Canada; 4 ISAGE (Indigenous Sovereignty, Autonomy, Governance & Ethics), Hanmer, Ontario, Canada; 5 Lawrence Bloomberg Faculty of Nursing, University of Toronto, Toronto, Ontario, Canada

## Abstract

**Importance:**

Massive volumes of health data flow to commercial data brokers worldwide, yet little empirical research has examined how this industry functions and the implications for patients.

**Objective:**

To describe and analyze the primary care medical record industry in Canada and its data collection and commercialization practices.

**Design, Setting, and Participants:**

This qualitative study of the Canadian primary care health data industry used situational analysis, a grounded theory methodology. Data sources included semistructured interviews of individuals affiliated with the commercial health data industry from May 2022 to May 2023 and publicly available documents. Data were analyzed from May 2022 to May 2024.

**Main Outcomes and Measures:**

Individual semistructured interviews and relevant publicly available documents were analyzed to gain an understanding of data collection and commercialization practices in the primary care record industry. The analysis involved a continuous and iterative process of data collection and analysis, theoretical sampling, data-driven coding, and creation of theoretical concepts.

**Results:**

A total of 19 interviews were conducted and 22 documents were sampled. Study participants described the primary care medical record industry in Canada as consisting of complex reciprocal relationships between commercial health data brokers, physicians, for-profit chains of primary care clinics, and pharmaceutical companies. In an emerging vertically integrated business model, the data broker brought the primary care clinics and physicians in house as a clinical subsidiary, thus obtaining more control over clinical practices. Participants understood the primary care medical record industry as having potential to transform patient care, but—because of financial considerations—also tied to pharmaceutical industry interests. According to participants, patients were not involved in decisions related to how their records were collected and used.

**Conclusions and Relevance:**

This qualitative study found that each entity within the Canadian primary care medical record industry contributes to, and benefits from, the conversion of patient medical records into commercial assets. The industry’s activities reflect the pharmaceutical companies’ interests. Patients are notably absent from decision-making; thus, the industry’s activities may not reflect their values or interests.

## Key Points

**Question:**

How does the primary care medical record industry in Canada function?

**Findings:**

This qualitative study including 19 interviews with industry employees, consultants, and contractors, as well as academics, found that the medical record industry consists of complex reciprocal relationships between commercial health data brokers, physicians, for-profit chains of primary care clinics, and pharmaceutical companies, where each entity contributes to and benefits from transforming patient medical records into commercial assets. In a vertically integrated model, the data broker brought the primary care clinics in house as a subsidiary, obtaining more control over clinical practices.

**Meaning:**

These findings suggest that the primary care medical record industry’s activities are likely to further the pharmaceutical industry’s influence over care.

## Teaser

This qualitative study describes the data collection and commercialization practices of the primary care medical record industry in Canada and the implications for patients.

## Introduction

Commercial data brokers—for-profit companies that collect and monetize personal information—have started acquiring patient medical records from primary care clinics.^1^ For example, IQVIA, the world’s largest health data broker, claims to have deidentified primary care patient medical records from many countries around the world including the US, Belgium, France, Germany, Spain, the UK, and Canada.^2,3^ Deidentification removes patient identifiers, and manipulates quasi-identifiers to lower, but not eliminate, the risk of reidentification.^4,5^

Commercial data brokers acquiring patient data is not new. Data brokers have been purchasing deidentified patient prescription data from pharmacies for more than 50 years.^6^ The data brokers monetize prescription data primarily by conducting analytics for the pharmaceutical industry.^7^ Pharmaceutical companies use the data for a variety of purposes including to plan and assess marketing campaigns, demonstrate safety and efficacy for regulators, determine burden of disease, understand physician prescribing habits, and identify physician opinion leaders.^8,9,10^ Governmental agencies and academics also use the data, largely for research and health system improvement.^11,12,13^

Primary care medical records, however, differ from prescription datasets; they are repositories of large amounts of health information collected from primary care visits and across the health system.^14,15,16^ For example, IQVIA claims that its Canadian primary care datasets include deidentified patient-level longitudinal information on diagnoses, treatments, billings, laboratory tests, diagnostics, vaccines, and specialist appointments dating back to 2010.^17^ The additional information may permit more robust analyses and health system improvements but may have unintended consequences such as greater risks to privacy.^18^

Despite the massive volumes of medical records flowing to commercial data brokers worldwide^2,3^ and the growing concern from the public, scholars, and media,^8,19,20,21,22,23^ little empirical research has examined how this industry functions and the implications for patients.^24,25,26,27^ Thus, in this study we aimed to explore the primary care medical record industry in the Canadian context and the implications of these activities for patients, communities, and society.

## Methods

### Design

This qualitative study was informed by situational analysis, a grounded theory methodology that helps generate a conceptual understanding of a complex social situation.^65^ We collected and triangulated 2 forms of qualitative data: individual semistructured interviews and relevant publicly available documents. We received ethics approval from the Women’s College Hospital research ethics board. Participants provided written informed consent prior to starting the interview. We report findings according to the Standards for Reporting Qualitative Research (SRQR) reporting guideline.^28^

### Setting

Most essential primary care services in Canada are fully funded by provincial or territorial public payers.^29,30,31^ Primary care physicians are largely self-employed practitioners and bill the provincial payer for the costs of running their practices. Some work individually in solo practices and others work in collaboratives with other physicians and health care clinicians, or increasingly, as contractors for for-profit companies.^32^ Provincial and territorial medical self-regulators govern physicians’ activities.^33^

### Sampling

We used theoretical sampling^34^ to identify documents and recruit individuals who, based on our initial conceptual understanding of the industry, were likely to be information-rich sources. We conducted structured internet searches and identified 2 commercial data brokers that offered third parties access to Canadian primary care patient medical records, each claiming to have access to between 1 and 2 million records. Through these internet searches, we also identified documents describing the data brokers’ activities. We used information in the public domain to recruit individuals employed by the data broker (and affiliated entities) who were involved in the collection and monetization of patient data. As the project progressed, we recruited based on our evolving analysis.^34^

### Data Collection

One investigator (L.V.) conducted semistructured telephone interviews to explore individuals’ experiences with the primary care medical record industry’s data collection and monetization practices, the flow of data and capital, and the potential benefits and risks for patients, communities, and society (eAppendix 1 in Supplement 1). The interview guide was modified throughout the process to reflect the ongoing data analysis. A professional transcriptionist transcribed the interviews. We removed all identifying information from transcripts and refer to participants by their role.

### Data Analysis

Data data collection and analysis proceeded simultaneously.^35,36^ Two investigators (L.V. and S.M.S.) conducted preliminary line-by-line coding of each data item, seeking to make “fundamental processes explicit [and] render hidden assumptions visible.”^37^ We relied on documents to develop our initial conceptual maps and to direct sampling of participants. As the analysis progressed, we used documents to supplement information gleaned from the interviews to corroborate findings, fill in gaps, and address conflicting information. We continually and iteratively compared data with previously collected data (constant comparative method), recorded interpretations and analytic directions in memos, and adjusted codes and categories as needed.^37^ Two investigators (L.V. and S.M.S.) produced and analyzed situational maps to understand the elements in the industry and their interrelationships.^38^ Using the maps, preliminary codes, and memos, we identified the more significant codes,^37^ which were used produce larger categories.^35,36^ We used the categories, maps, memos, and rereadings of the data, to generate theoretical concepts—higher-level categories that subsume and organize the categories (eAppendix 2 in Supplement 1). We completed data collection and analysis once we reached theoretical saturation—when newly collected data were no longer leading to significant changes in our conceptual understanding of the situation. The research team met regularly during data collection and analysis to review and provide feedback. NVIVO software version 12 (Lumivero) was used to code data from May 2022 to May 2024.

## Results

We interviewed 19 participants affiliated with the Canadian primary care medical record industry between May 2022 and May 2023 (Table 1). We sampled 22 publicly available documents from 7 types of sources (eg, data broker websites, government websites, and academic papers) produced between 2015 and 2023 and we accessed these documents between May 2022 and May 2024 (Table 2).

**Table 1.** Description of Participants (Roles)^a^

Role	Participants, No.
Academics	2
Community researcher with expertise in Indigenous health	1
Consultants	2
Data broker employees	8
Electronic medical record vendor employee	1
Government employees	2
Pharmaceutical company employee	1
Physicians (contractors working at for-profit chains of primary care clinics)	2

^a^ Some participants had more than 1 role but, to protect their privacy, they are described in this study by only their most relevant role and using gender neutral pronouns.

**Table 2.** Descriptions of Documents

Document No.	Sector	Category	Location	Description	Year published
1	Industry	Promotional materials	Data broker website	Provide quotes from physicians describing clinical activities	2021
2	Media	Press release	News wire network	Describes data broker activities	2022
3	Industry	Promotional materials	Data broker website	Lists of pharmaceutical industry partners	2021
4	Industry	Investor materials	Data broker website	Describes data broker activities	2021
5	Industry	Conference abstract	Trade conference website	Describes data broker activities	2019
6	Industry	Promotional materials	Data broker website	Describes data broker activities	2020
7	Industry	Conference presentation	Trade conference website	Describes data broker activities	2021
8	Industry	Promotional materials	Data broker website	Market study	2022
9	Industry	Promotional materials	Data broker website	Describes primary care datasets	2021
10	Industry	Investor materials	Data broker website	Describes data broker activities	2022
11	Industry	Investor materials	Data broker website	Provides a preliminary prospectus for initial public offering	2020
12	Industry	Privacy policy	Data broker website	Addresses the collection and use of patient data	2015
13	Media	Press release	Media website	Describes data broker activities	2023
14	Media	Newspaper article (sponsored)	Media website	Describes data broker activities	2021
15	Academic	Journal article	Medical journal	Describes primary care datasets	2022
16	Industry	Promotional materials	Data broker website	Describes data broker activities	2022
17	Media	Newspaper article	Media website	Describes how patient data are commercialized in Canada	2019
18	Government	Regulator’s ruling	Government website	Ruling (Personal Health Information Protection Act decision 175)	2023
19	Industry	Promotional materials	Primary care clinic chain website	Describes clinical activities	2024
20	Industry	Promotional materials	Data broker website	Describes activities of analytics subsidiary	2024
21	Industry	Promotional materials	Data broker website	Describes clinical activities	2024
22	Industry	Investor information	Investment bank website	Describes data broker activities	2021

### The Primary Medical Record Industry in Canada: An Overview

**Figure 1.**
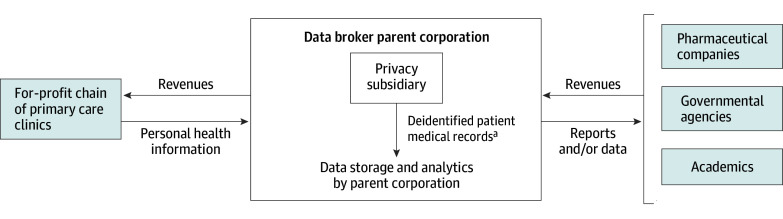
Conventional Model of Data Acquisition and Commercialization ^a^Personally identifying information are removed to make the risk of reidentification low.

**Figure 2.**
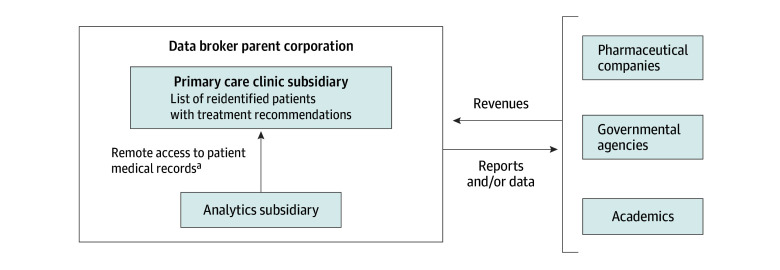
Vertically Integrated Model of Data Acquisition and Commercialization ^a^Names replaced with numeric pseudonym.

Participants described how data brokers acquired primary care patient medical records through 2 approaches. In the conventional model, the data broker acquired records from another company, a for-profit chain of primary care clinics (Figure 1). A subsidiary of the data broker deidentified the data prior to data storage and analysis. In an emerging vertically integrated model (Figure 2), a commercial data broker gained access to primary care patient medical records directly through a clinical subsidiary, which operated a chain of primary care clinics. The data broker replaced patients’ names with a numeric pseudonym prior to analysis. In the conventional model, data brokers monetized the assets primarily by conducting analytics for pharmaceutical companies and, in the vertically integrated model, by identifying and targeting patients who may qualify for a pharmaceutical company’s drug through algorithms and subsequent messaging to physicians.

### Complex Reciprocal Relationships

Participants described how the primary care medical record industry functions and how corporate interests are reflected in the day-to-day behaviors of clinic managers and physicians. The participants provide insight into patient involvement and give their views on the risks and benefits of the nascent industry. Study participants also described how data brokers, physicians, for-profit chains of primary care clinics, and pharmaceutical companies contributed to, and benefitted from, the conversion of patient medical records into revenue-generating assets.

#### For-Profit Chains

The for-profit chains of clinics provided access to large collections of patients’ primary care medical records. These databases, according to a data broker’s investor report, could potentially generate hundreds of millions of dollars in revenue for the data broker (document 4). The clinics benefitted from opportunities to participate in clincial research (document 19) and by gaining a revenue stream (academic researcher interview) and opportunities to make money (interview with physician working on contract at a clinic operated by a data broker). Relationships with these for-profit clinic chains were attractive to data brokers, in contrast with solo or group practices, because they had access to data at scale. An academic researcher explained how extracting, deidentifying, and preparing data for analysis was a labor-intensive process and going clinic by clinic was not a scalable solution, “but when you have one chain that owns 40, 50, 60, 100 clinics or whatever it may be, that’s an easier business arrangement to make.”

#### Data Brokers

Data brokers contributed by turning large volumes of unstructured data into analytical-ready, (document 16) rich datasets with detailed information about “what drugs [patients] are taking and what kind of comorbidities they have and the regular labs and what they have and whether they smoke and whether they do this and that” (academic researcher interview). The data brokers also contributed by conducting analytics for clients; clients generally wanted reports and answers to questions rather than the raw data (data broker employee interviews). Finally, in the vertically integrated business model, the data broker offered the ability to identify and directly target patients (via messaging to physicians) with interventions.

#### Pharmaceutical Companies

In the conventional business model, pharmaceutical companies benefitted by gaining access to analytics on primary care patients and on physician behaviors. A pharmaceutical company employee described how the primary care data were used to show regulators that there was a health need in terms of “how many patients are out there, how many eligible patients would be out there,” and once the drug was on the market, to demonstrate drug efficacy or safety. The primary care datasets were also used for business insights, defined by the pharmaceutical company employee as “things that can be gleaned that would be helpful to the business itself.” These insights, according to the pharmaceutical company employee, were directed toward identifying the activities pharmaceutical companies take to ensure that “all people who need access to the drug get it.” The pharmaceutical company employee explained, “If you’re the [pharmaceutical] company that puts out a biologic” and there are “four [physicians] at an institution and [three of] them prescribe biologics, but one doesn’t, would you not want to understand why?” According to the pharmaceutical company employee, “good decision-making is guided by data” and “most [pharmaceutical companies], if not all, are doing these kinds of business and clinical insights from administrative data.”

In the vertically integrated model, data brokers offered sponsorship arrangements to pharmaceutical companies whereby the data broker would identify patients, through their medical records, who might be eligible for the sponsor’s treatment. A data broker employee explained how this worked. Companies sponsored the data broker to create or operationalize an algorithm to identify patients with a particular health condition. Then, once a physician consented to participate in the initiative, the data broker ran the algorithms on the patients’ medical records (identified by numeric pseudonyms not names) and generated the list of relevant patients. A data broker employee provided a list of reidentified patients to the physician along with treatment recommendations based on published guidelines. The data broker employee emphasized that the treatment recommendations did not include the names of a specific drug. However, according to the data broker employee, for many of the conditions, “there’s probably just one drug…no other options” for treatment and, as such, the pharmaceutical company was counting on the patient getting their drug.

#### Physicians

Physicians also played important roles in the industry. In their role as contractors at for-profit clinics, they provided medical care as well as gave their consent to allow data brokers to access the patient medical records (data broker employee interviews; document 12). In the vertically-integrated model, they were also tasked with reviewing the list of patients the algorithm generated and reaching out to the patients for further assessment (data broker employee interviews and physician interviews). In exchange for their time spent reviewing the data broker’s treatment recommendations, physicians received financial compensation from the data broker (data broker employee interview). A physician working on contract at a clinic operated by a data broker explained, “I think I told you about the offer to pay us to review this list of patients with [the data broker employee]. So that would be one way of being financially compensated for your extra time reviewing those files with them.”

Physicians also benefitted, according to a data broker employee, by gaining access to the algorithms, characterized by the employee as decision support tools. In the data broker employee’s view, these tools alerted physicians to the patients who may benefit from new drug treatments even if the “drug rep hadn’t been to [the physicians] yet” to inform them about new treatments. A physician working on contract at a clinic operated by the data broker agreed, describing the experience as a win for all because it helped patients access treatment, assisted physicians in providing better care, and provided financial returns to companies.

### Tied to the Interests of the Pharmaceutical Industry

Study participants described how in both models, pharmaceutical companies were the data brokers’ primary clients. As the primary clients, the pharmaceutical company interests were made explicit in the day-to-day running of clinic operations. Participants generally felt that the benefits of pharmaceutical industry involvement outweighed the risks.

#### The Primary Client

In the conventional model, the pharmaceutical industry was the commercial data broker’s primary client (data broker employee interviews). According to a data broker employee, “over 90% of the requests [for the primary care datasets] came from pharmaceutical companies.” Other clients included governments (data broker and consultant interviews) as well as academics (academic researcher interview). Likewise, in the vertically-integrated model, the pharmaceutical industry was the data broker’s primary client. Although the data broker had hundreds of algorithms to identify health conditions according to 2 data broker employees, the data broker prioritized algorithms with a sponsor, because the sponsor “pays the team.” These sponsors were almost always pharmaceutical companies. A data broker employee recalled only one instance where the data broker ran an algorithm that was not sponsored by a pharmaceutical company. A physician reported that their experience working on contract at a for-profit chain of primary care clinics, aligned. During their time at the clinic, the physician reported, “there were two conditions [the data broker was screening for].” Each condition was associated with “one specific drug that has been called out within the last few years” in treatment guidelines. Further, a data broker employee confirmed to the physician that pharmaceutical companies were involved. The physician also noted “the conditions have a specific medication, most of them recent and expensive medication that could be used.” As such, these sponsorship arrangements created incentive structures to promote or prompt the use of specific drugs within the clinical practice.

#### Day-to-Day Clinical Activities

The interests of the pharmaceutical companies, as the data brokers’ primary clients, were visible in the day-to-day activities and responsibilities of clinic managers and physicians. According to a data broker employee, the data broker had to identify a minimum number of clinic patients with the target condition, or the “pharma company…would probably cut down [sponsorship] the next quarter.”

The clinic managers operationalized these arrangements by requesting that physicians participate in the initiative. For example, a physician described how the clinic managers asked the physicians to participate in sharing patient medical records: “The first time the clinic [managers] said, ‘it will be absolutely up to you whether you want to do it’… But actually, it was slightly different. We didn’t have to, but it was put like this, ‘Well, if someone wants to contact you, please give them a date when you could talk.’ It’s your choice, but it was a bit more matter of fact then.”

After a physician received the list of candidate patients and treatment recommendations from the data broker employee, they were asked by clinic managers to check “whether this patient would be a good candidate for that specific medication and/or refer to a specialist for consultation of the use of this specific medication.” This physician’s experience indicates, that although the official treatment recommendations made by the company do not include names of specific drugs (data broker employee interview), clinic managers may convey this information to physicians.

#### Perceived Patient Benefits Outweighing the Risks From Pharmaceutical Industry Involvement

Study participants believed the primary care medical record industry had the potential to improve patient care, largely by providing increased access to pharmaceutical treatments (data broker employee, Indigenous researcher, physician, and academic researcher interviews). These treatments were likely to be new, on-patent drugs, according to a physician working on contract at a clinic operated by a data broker, because pharmaceutical companies were unlikely to promote older off-patent drugs. A data broker employee explained how the industry helped patients that the algorithm identified as possibly having specific health conditions by giving them access to treatment: “We would send that short-list to the physician saying, ‘This patient is exhibiting these symptoms, they have not been well for a while and nothing is working, this patient might have this [health condition].’ And then the doctor would say, ‘Great, let me do the work up for that specific disease.’ So many patients have benefitted from this approach.”

This data broker employee espoused a highly instrumental approach, concluding, “It doesn’t matter whether a [pharmaceutical company] has a relationship with the [data broker and medical clinics] or not, as long as the final output is a patient getting the treatment.” Further, the health or ethical risks of this approach were acceptable in the views of some participants because they held physicians accountable for patient care. Another data broker employee argued that even though the pharmaceutical industry was involved, physicians were ultimately “responsible for the patients’ care.” The pharmaceutical companies raised awareness, but physicians made the treatment decisions (data broker employee interview). This view positions physicians as independent arbiters, who weigh the information and are responsible for the patient care decisions, discounting how physicians may be affected by their relationships with clinic managers, data brokers, and pharmaceutical industry representatives.

### Patients: Absent From Decision-Making

Participants were not aware of any attempts to include patients in decisions regarding the collection and use of their medical records (physician and data broker employee interviews). According to a data broker employee, no one sought consent from patients to access and use their records. Another data broker employee described how physicians made the decisions for patients and their records. Similarly, a data broker privacy statement described how the data broker sought physician consent to access patient medical records but made no mention of patient consent (document 12). A physician described patients’ data as “snatched away,” noting, “It’s patient’s data but how is it that these companies even can own the data? Why is it even legal? Why are they allowed to do anything with the data? … I don’t see how it should even be legal to provide this information [to commercial data brokers].”

A government employee had concerns that data brokers were using the medical records without a social license, meaning without widespread public support for data uses and users. The government employee explained that people living in Canada supported sharing their medical records with the “government to improve the efficacy of the health system itself,” and with academic researchers but say “no, no, no” to sharing data with the private sector.

The concept of a social license for data collection and use is further complicated when accounting for the experiences of Indigenous communities. For example, a community researcher with expertise in Indigenous health explained, “The problem has always been that there’s distrust, and especially for First Nation people, about what happens to your personal information. We [as a society] don’t have a system [for managing personal information] that’s perfect…There’s a lot of work there, to make sure that individuals, like myself, feel that my personal information is being protected.”

Further, according to the community researcher, the companies should be following the Ownership, Control, Access and Possession (OCAP) principles for First Nations data, which “means that each First Nation…has the right to be able to say what happens to the individual community members’ data, health data.” However, study participants were not aware of attempts to seek consent from Indigenous nations and communities to access and use medical records from community members.

## Discussion

This qualitative study found that the primary care medical record industry in Canada consists of complex reciprocal relationships between commercial health data brokers, physicians, for-profit chains of primary care clinics, and pharmaceutical companies. Each entity contributes to, and benefits from, the conversion of patient medical records into commercial assets. In an emerging vertically integrated model, the data broker brings the primary care clinics in house as a clinical subsidiary, tightening the relationships, gaining increased control over clinical activities and the ability to identify and target patients (via physicians) with clinical interventions. Study participants understood the primary care medical record industry as having potential to transform patient care, but—because of financial considerations—tied to pharmaceutical industry interests. Despite potential effects on their care and privacy, patients and Indigenous communities do not appear to be included in decisions related to how their records are collected and used.

Electronic medical records and associated data technologies have driven a seismic shift in health systems and society, producing new interrelationships and interactions and disrupting the old. The technologies enable important research and health system improvements^39^ but, as demonstrated in our study, are used to give the pharmaceutical industry increased influence over primary care.^24^ The vertically integrated model offers pharmaceutical companies a powerful additional mechanism to increase use of their drug products while also allowing them to increase market control and ward off competition.^40^ Problematically, pharmaceutical industry influence is likely to lead to an increased focus on health conditions for which pharmaceutical companies market treatments and skew resources toward heavily promoted prescription drug treatments.^24,41,42^ These promoted treatments are more likely to be branded, on-patent, and newer, with less established safety profiles, and higher costs, compared with cost-effective generic counterparts.^43,44,45,46^

Our analysis indicates that the primary care medical record industry in Canada does not seek patient or community involvement in how data are collected and used in a substantive way. Participants were not aware of any mechanisms to seek patient consent or input. Instead, companies appeared to seek out physician consent to access patient records, reflecting Canadian privacy legislations that designate physicians as the data custodians for patient medical records.^47,48,49,50^ However, as patients seek care through medical clinics owned by large for-profit corporations, the balance of power shifts and physicians may no longer be able to put patient needs first. Instead, physicians may, consciously or subconsciously, feel an obligation to act to meet the objectives of the larger corporation, a powerful organized entity.^51,52,53,54,55,56,57,58^

If patient medical records are deidentified, these practices may comply with a recent ruling from the privacy commissioner of Ontario stipulating, that under current privacy legislation, data custodians (in this case, the companies) only need to inform patients, not seek their consent, to deidentify and use their data.^59^ If patient data are not deidentified, as in the vertical model, the legality is unclear. Either way, these approaches do not appear to align with patient values. People have lower levels of trust in commercial entities compared with medical organizations and public research bodies; they are often reluctant to share their data, identified or not, and, at minimum, want public oversight and proof that data are being used for the public good.^21,60,61,62,63,64,65,66^ Further, because participants were not aware of attempts to implement Indigenous data sovereignty principles, the industry’s approach to gathering and using data appears to violate Indigenous Peoples’ right to have control over their information.^67,68,69^

To address these findings, medical regulators should provide guidance to physicians who contract at clinics that supply data to commercial data brokers. Privacy regulators should assess the legality of the business activities in the vertically integrated model; policymakers should create legislations that promote transparency, community and public governance of data, and Indigenous data sovereignty.

### Limitations

This qualitative study has limitations. We used interviews and documents to provide a rich description and analysis of the primary care medical record industry. We, however, were limited to publicly available documents; proprietary documents would have provided additional information about the complex situation.^70^ Future work would also benefit from input from those affected by the activities of this industry including patients, members of structurally marginalized groups, and Indigenous communities.

## Conclusions

The entities involved in the primary care medical record industry in Canada—chains of for-profit primary care clinics, physicians, commercial data brokers, and pharmaceutical companies—work together to convert patient medical records into commercial assets. These assets are largely used to further the interests of the pharmaceutical companies. As a result, the industry has implications for patient care and health system costs. Changes to legislation and regulation may help to ensure that data use reflect people’s interests and values.

## Notes

Supplement 1. **eAppendix 1.** Interview Guide **eAppendix 2.** Concept, Category and Code Descriptions

Supplement 2.
Data Sharing Statement

## References

[1] Singer N, Wakabayashi D. Google to store and analyze millions of health records. *New York Times*. Published November 12, 2019. Accessed June 3, 2024. https://www.nytimes.com/2019/11/11/business/google-ascension-health-data.html

[2] IQVIA. Real world and health data sets. Published 2023. Accessed August 30, 2023. https://web.archive.org/web/20230830171411/https://www.iqvia.com/solutions/real-world-evidence/real-world-data-and-insights

[3] IQVIA. UK longitudinal patient data. Accessed April 1, 2025. https://www.iqvia.com/library/fact-sheets/uk-longitudinal-patient-data

[4] Information and Privacy Commissioner of Ontario. De-identification guidelines for structured data. Published June 2016. Accessed March 20, 2025. https://www.ipc.on.ca/sites/default/files/legacy/2016/08/Deidentification-Guidelines-for-Structured-Data.pdf

[5] Cavoukian A, El Emam K. Dispelling the myths surrounding de-identification: anonymization remains a strong tool for protecting privacy. Information and Privacy Commissioner of Ontario. Published June 2011. Accessed March 20, 2025. https://www.csri.info/wp-content/uploads/2012/08/anonymization.pdf

[6] Tanner A. Our Bodies, Our Data: How Companies Make Billions Selling Our Medical Records. Beacon Press; 2017.

[7] IQVIA. QuintilesIMS is now IQVIA. Published November 6, 2017. Accessed March 20, 2025. https://www.iqvia.com/newsroom/2017/11/quintilesims-is-now-iqvia

[8] Tanner A. How data brokers make money off your medical records. *Scientific American*. Published February 1, 2016. Accessed June 1, 2020. https://www.scientificamerican.com/article/how-data-brokers-make-money-off-your-medical-records/

[9] QuintilesIMS. IMS Xponent: clarity and confidence in a complex world. Published 2017. Accessed July 19, 2018. https://web.archive.org/web/20180619105940/http://www.imsbrogancapabilities.com:80/pdf/healthcare-xponent.pdf

[10] Johnson I. Leveraging key thought leaders to drive brand growth. Canadian Pharmaceutical Marketing. Published February 2011. Accessed February 12, 2021. https://web.archive.org/web/20160705083905/http://imsbrogancapabilities.com/pdf/consulting-influential-thought-leader.pdf

[11] McGirr A, Bourgoin T, Wortzman M, Millson B, McNeil SA. An early look at the second dose completion of the recombinant zoster vaccine in Canadian adults: a retrospective database study. Vaccine. 2021;39(25):3397-3403. doi:10.1016/j.vaccine.2021.04.053 34001346

[12] Stankus V, Hemmelgarn B, Campbell NRC, Chen G, McAlister FA, Tsuyuki RT. Reducing costs and improving hypertension management. Can J Clin Pharmacol. 2009;16(1):e151-e155. 19193969

[13] Government of Canada. NPDUIS source materials. Published 2018. Accessed January 21, 2021. https://web.archive.org/web/20210121191756/https://www.pmprb-cepmb.gc.ca/view.asp?ccid=1415&lang=en

[14] Gentil ML, Cuggia M, Fiquet L, . Factors influencing the development of primary care data collection projects from electronic health records: a systematic review of the literature. BMC Med Inform Decis Mak. 2017;17(1):139. doi:10.1186/s12911-017-0538-x 28946908 PMC5613384

[15] Shi L. The impact of primary care: a focused review. Scientifica (Cairo). 2012;2012:432892. doi:10.6064/2012/432892 24278694 PMC3820521

[16] IMS Health Brogan. IMS health: unlocking the value of EMR data for advanced research and analysis, better health metrics, and product innovation. QuintilesIMS. Published 2017. Accessed December 22, 2020. https://web.archive.org/web/20210216165758/https://privacy-analytics.com/wp-content/uploads/dlm_uploads/2020/06/IMS-Brogan-Case-Study.pdf

[17] IQVIA. EMR primary care data. Published November 1, 2021. Accessed August 30, 2023. https://www.iqvia.com/locations/canada/library/fact-sheets/emr-primary-care-data

[18] Spithoff S, Grundy Q. Commercializing personal health information: a critical qualitative content analysis of documents describing proprietary primary care databases in Canada. Int J Health Policy Manag. 2023;12(1):6938. doi:10.34172/ijhpm.2023.6938 37579404 PMC10461871

[19] Graham M. Data for sale: trust, confidence and sharing health data with commercial companies. J Med Ethics. 2023;49(7):515-522. doi:10.1136/medethics-2021-107464 34330796 PMC10359563

[20] Ghafur S, Van Dael J, Leis M, Darzi A, Sheikh A. Public perceptions on data sharing: key insights from the UK and the USA. Lancet Digit Health. 2020;2(9):e444-e446. doi:10.1016/S2589-7500(20)30161-8 32838250 PMC7380931

[21] Paprica PA, de Melo MN, Schull MJ. Social licence and the general public’s attitudes toward research based on linked administrative health data: a qualitative study. CMAJ Open. 2019;7(1):E40-E46. doi:10.9778/cmajo.20180099 30718354 PMC6375226

[22] American Medical Association. Patient perspectives around data privacy. Published 2022. Accessed March 20, 2025. https://www.ama-assn.org/system/files/ama-patient-data-privacy-survey-results.pdf

[23] Butler E. Should we trust big tech with our health data? BBC. Published July 14, 2021. Accessed September 23, 2024. https://www.bbc.com/news/business-57817804

[24] Mulinari S, Ozieranski P. Capitalizing on transparency: commercial surveillance and pharmaceutical marketing after the Physician Sunshine Act. Big Data Soc. Published online February 9, 2022. doi:10.1177/20539517211069631

[25] Ebeling MFE. Healthcare and Big Data: Digital Specters and Phantom Objects. 1st ed. Palgrave Macmillan; 2016.

[26] Friedman AB, Merchant RM, Maley A, . Widespread third-party tracking on hospital websites poses privacy risks for patients and legal liability for hospitals. Health Aff (Millwood). 2023;42(4):508-515. doi:10.1377/hlthaff.2022.01205 37011312 PMC11145977

[27] Feathers T, Palmer K, Fondrie-Teitler S. “Out Of control”: dozens of telehealth startups sent sensitive health information to big tech companies. The Markup. Published December 13, 2022. Accessed September 24, 2023. https://themarkup.org/pixel-hunt/2022/12/13/out-of-control-dozens-of-telehealth-startups-sent-sensitive-health-information-to-big-tech-companies

[28] O’Brien BC, Harris IB, Beckman TJ, Reed DA, Cook DA. Standards for reporting qualitative research: a synthesis of recommendations. Acad Med. 2014;89(9):1245-1251. doi:10.1097/ACM.0000000000000388 24979285

[29] Bryant T. Health Policy in Canada. 2nd ed. Canadian Scholars; 2016.

[30] Martin D, Miller AP, Quesnel-Vallée A, Caron NR, Vissandjée B, Marchildon GP. Canada’s universal health-care system: achieving its potential. Lancet. 2018;391(10131):1718-1735. doi:10.1016/S0140-6736(18)30181-8 29483027 PMC7138369

[31] Hutchison B, Levesque JF, Strumpf E, Coyle N. Primary health care in Canada: systems in motion. Milbank Q. 2011;89(2):256-288. doi:10.1111/j.1468-0009.2011.00628.x 21676023 PMC3142339

[32] Hedden L, McGrail K. The best defence is a good offence: Ensuring equitable access to primary care in Canada. Healthc Manage Forum. 2023;36(5):293-298. doi:10.1177/08404704231182260 37500185 PMC10448912

[33] Adams TL. Health professional regulation in historical context: Canada, the USA and the UK (19th century to present). Hum Resour Health. 2020;18(1):72. doi:10.1186/s12960-020-00501-y 33076923 PMC7572238

[34] Patton MQ. Qualitative Research & Evaluation Methods: Integrating Theory and Practice. 4th ed. SAGE Publications, Inc; 2014.

[35] Clarke AE, Friese C, Washburn RS. Situational Analysis: Grounded Theory After the Interpretive Turn. 2nd ed. SAGE Publications, Inc; 2017.

[36] Corbin J, Strauss A. Basics of Qualitative Research. SAGE; 2015.

[37] Charmaz K. Constructing Grounded Theory. 2nd ed. SAGE Publications Ltd; 2014.

[38] Clarke AE, Friese C, Washburn R. Situational Analysis: Grounded Theory After the Interpretive Turn. 2nd ed. Sage Publications; 2017.

[39] Thandi M, Wong ST, Aponte-Hao S, . Strategies for working across Canadian practice-based research and learning networks (PBRLNs) in primary care: focus on frailty. BMC Fam Pract. 2021;22(1):220. doi:10.1186/s12875-021-01573-y 34772356 PMC8590340

[40] Khan LM. Amazon’s antitrust paradox. The Yale Law Journal. Published 2017. Accessed March 20, 2025. https://www.yalelawjournal.org/pdf/e.710.Khan.805_zuvfyyeh.pdf

[41] Spurling GK, Mansfield PR, Montgomery BD, . Information from pharmaceutical companies and the quality, quantity, and cost of physicians’ prescribing: a systematic review. PLoS Med. 2010;7(10):e1000352. doi:10.1371/journal.pmed.1000352 20976098 PMC2957394

[42] Sismondo S. Ghost-Managed Medicine: Big Pharma’s Invisible Hands. Mattering Press; 2018. doi:10.28938/9780995527775

[43] Lasser KE, Allen PD, Woolhandler SJ, Himmelstein DU, Wolfe SM, Bor DH. Timing of new black box warnings and withdrawals for prescription medications. JAMA. 2002;287(17):2215-2220. doi:10.1001/jama.287.17.2215 11980521

[44] Brody H, Light DW. The inverse benefit law: how drug marketing undermines patient safety and public health. Am J Public Health. 2011;101(3):399-404. doi:10.2105/AJPH.2010.199844 21233426 PMC3036704

[45] Onakpoya IJ, Heneghan CJ, Aronson JK. Post-marketing withdrawal of anti-obesity medicinal products because of adverse drug reactions: a systematic review. BMC Med. 2016;14(1):191. doi:10.1186/s12916-016-0735-y 27894343 PMC5126837

[46] Lexchin J. The relation between promotional spending on drugs and their therapeutic gain: a cohort analysis. CMAJ Open. 2017;5(3):E724-E728. doi:10.9778/cmajo.20170089 28912143 PMC5621942

[47] Government of Ontario. Personal Health Information Protection Act, 2004, S.O. 2004, c. 3, Sched. A. Updated November 30, 2024. Accessed February 10, 2025. https://www.ontario.ca/laws/statute/04p03

[48] Nova Scotia Legislature. Personal health information act. Published December 10, 2010. Accessed March 20, 2025. https://nslegislature.ca/legc/bills/61st_2nd/3rd_read/b089.htm

[49] Information and Privacy Commissioner of Ontario. Frequently asked questions: personal health information protection act. Published September 2015. Accessed September 12, 2019. https://www.ipc.on.ca/wp-content/uploads/2015/11/phipa-faq.pdf

[50] Province of Alberta. Health information act. Published 2001. Updated December 20, 2024. Accessed March 20, 2025. https://kings-printer.alberta.ca/documents/Regs/2001_070.pdf

[51] Starr P. The Social Transformation of American Medicine: The Rise of a Sovereign Profession and the Making of a Vast Industry. 2nd ed. Basic Books; 2017.

[52] Zhu JM, Rooke-Ley H, Fuse Brown E. A doctrine in name only—strengthening prohibitions against the corporate practice of medicine. N Engl J Med. 2023;389(11):965-968. doi:10.1056/NEJMp2306904 37694885

[53] Schmunk R, King A, Ward L. Corporate pressure led Shoppers Drug Mart staff to bill for unnecessary medication reviews, pharmacists say. CBC News. Published February 28, 2024. Accessed June 4, 2024. https://www.cbc.ca/news/canada/ontario-medcheck-shoppers-drug-mart-pressure-1.7126811

[54] Talbot SG, Dean W. Physicians aren’t “burning out.” They’re suffering from moral injury. STAT. Published July 26, 2018. Accessed June 4, 2024. https://www.statnews.com/2018/07/26/physicians-not-burning-out-they-are-suffering-moral-injury/

[55] Grumbach K, Osmond D, Vranizan K, Jaffe D, Bindman AB. Primary care physicians’ experience of financial incentives in managed-care systems. N Engl J Med. 1998;339(21):1516-1521. doi:10.1056/NEJM199811193392106 9819451

[56] Deom M, Agoritsas T, Bovier PA, Perneger TV. What doctors think about the impact of managed care tools on quality of care, costs, autonomy, and relations with patients. BMC Health Serv Res. 2010;10:331. doi:10.1186/1472-6963-10-331 21138576 PMC3016355

[57] Press E. The moral crisis of America’s doctors. *New York Times*. Published June 15, 2023. Accessed June 4, 2024. https://www.nytimes.com/2023/06/15/magazine/doctors-moral-crises.html

[58] Salisbury H. Helen Salisbury: does it matter who owns general practice? BMJ. 2021;373(1026):n1026. doi:10.1136/bmj.n1026 33883173

[59] Khosseim P. Ripe for public debate: legal and ethical issues around de-identified data. IPC. Published May 17, 2022. Accessed September 6, 2022. https://www.ipc.on.ca/ripe-for-public-debate-legal-and-ethical-issues-around-de-identified-data/

[60] Stockdale J, Cassell J, Ford E. “Giving something back”: a systematic review and ethical enquiry into public views on the use of patient data for research in the United Kingdom and the Republic of Ireland. Wellcome Open Res. 2019;3:6. doi:10.12688/wellcomeopenres.13531.2 30854470 PMC6402072

[61] Kalkman S, van Delden J, Banerjee A, Tyl B, Mostert M, van Thiel G. Patients’ and public views and attitudes towards the sharing of health data for research: a narrative review of the empirical evidence. *BMJ Journal of Medical Ethics*. 2019; 48(1):3-13. 10.1136/medethics-2019-105651 PMC8717474 31719155

[62] Grande D, Mitra N, Shah A, Wan F, Asch DA. Public preferences about secondary uses of electronic health information. JAMA Intern Med. 2013;173(19):1798-1806. doi:10.1001/jamainternmed.2013.9166 23958803 PMC4083587

[63] Aitken M, de St Jorre J, Pagliari C, Jepson R, Cunningham-Burley S. Public responses to the sharing and linkage of health data for research purposes: a systematic review and thematic synthesis of qualitative studies. BMC Med Ethics. 2016;17(1):73. doi:10.1186/s12910-016-0153-x 27832780 PMC5103425

[64] Ipsos MORI Social Research Institute. The one-way mirror: public attitudes to commercial access to health data. Published February 2016. Accessed July 16, 2019. https://www.ipsos.com/sites/default/files/publication/5200-03/sri-wellcome-trust-commercial-access-to-health-data.pdf

[65] Trinidad MG, Platt J, Kardia SLR. The public’s comfort with sharing health data with third-party commercial companies. Humanit Soc Sci Commun. 2020;7(1):1-10. doi:10.1057/s41599-020-00641-5 34337435 PMC8320359

[66] Willison DJ. Use of data from the electronic health record for health research—current governance challenges and potential approaches. Office of the Privacy Commisioner of Canada. Published March 2009. Accessed July 18, 2019. https://www.priv.gc.ca/en/opc-actions-and-decisions/research/explore-privacy-research/2009/ehr_200903/

[67] First Nations Information Governance Centre. Ownership, control, access and possession (OCAP^TM^): the path to first nations information governance. Published May 23, 2014. Accessed November 27, 2020. https://web.archive.org/web/20200909011418/https://fnigc.ca/sites/default/files/docs/ocap_path_to_fn_information_governance_en_final.pdf

[68] Carroll SR, Garba I, Figueroa-Rodríguez OL, . The CARE Principles for Indigenous Data Governance. Data Sci J. 2020;19(1):43. doi:10.5334/dsj-2020-043

[69] Inuit Tapiriit Kanatami. National Inuit strategy on research. Published 2018. Accessed March 20, 2025. https://www.itk.ca/wp-content/uploads/2018/04/ITK_NISR-Report_English_low_res.pdf

[70] Anderson SJ, McCandless PM, Klausner K, Taketa R, Yerger VB. Tobacco documents research methodology. Tob Control. 2011;20(Suppl_2)(suppl 2):ii8-ii11. doi:10.1136/tc.2010.041921 21504933 PMC3085001

