# Supplementary material for: The Primary Care Medical Record Industry in Canada and Its Data Collection and Commercialization Practices
Source: JAMA Netw Open. 2025 May 5;8(5):e257688. doi: 10.1001/jamanetworkopen.2025.7688 (PMC12053517; doi:10.1001/jamanetworkopen.2025.7688)
Supplement: Supplement 1. — eAppendix 1. Interview Guide eAppendix 2. Concept, Category and Code Descriptions [file jamanetwopen-e257688-s001.pdf]

Supplemental Online Content

Spithoff S, Vesely L, McPhail B, Row RK, Mogic L, Grundy Q. The primary care medical record industry in Canada and its data collection and commercialization practices. *JAMA Netw. Open*. 2025;8(4):e257688. doi:10.1001/jamanetworkopen.2025.7688

eAppendix 1. Interview Guide

eAppendix 2. Concept, Category and Code Descriptions

This supplemental material has been provided by the authors to give readers additional information about their work.

© 2025 Spithoff S et al. *JAMA Network Open*.

eAppendix 1. Interview Guide
This Interview guide is for individuals who responded to an email recruitment script and signed a consent form agreeing to participate in the interview.
Interviewers: Study qualitative researcher and principal investigator (Dr. Sheryl Spithoff)

Please Note: *This guide only represents the main themes to be discussed with the participants and as such does not include the various probes that may also be used. Non-leading prompts (in italics) will also be used, such as "Can you please tell me a little bit more about that?" and "What does that look like for you?" when probing a vague statement such as "I was active." Concrete examples will be asked for, regarding general descriptive statements that are made.*

Introduction
Thank you for agreeing to participate in this interview. My name is [name] and my role on this project is [describe role]. We are interviewing you to gain insight into the flow of de-identified primary care patient data in Canada from collection of data and creation of proprietary primary care datasets (**which we define as datasets owned or held by for-profit companies**), to end uses. In particular, we may ask you questions about your experiences working at an entity that collects, de-identifies or uses these proprietary primary care datasets. We will also seek your thoughts on the benefits and risks of proprietary primary care datasets owned or held by for-profit companies. Participation in this interview is voluntary. The interview should take approximately 30 to 50 minutes. I will audio record the interview for future data analysis. If you are not comfortable with the interview being audio recorded, we can take notes instead. All responses will be kept confidential and will only be shared with research team members. No identifying information, including information identifying your place of employment, will be included in our report. If the results of this study are published in articles or presented in public places your quotations (words that you say directly) may be used in reports or presentations but will be presented so as not to identify you. You will be referred to by a pseudonym and your name, name of your employer, or any other information that could identify you, will never be associated with anything you say. You may decline to answer any question or stop the interview at any time or take a break for any reason. Do you have any questions about what I have just explained?

Start recording
Interview questions

1. Without providing identifying or sensitive information, can you describe your current role?
    a. Please describe your current or past experience working with primary care patient data, in particular primary care datasets held or owned by for-profit companies?

2. Without providing identifying or sensitive information, can you tell me about [entity X]?

3. Based on your knowledge/experience, can you tell us about how for-profit companies get access to primary care medical records? How are the records de-identified?
        i. How do these for-profit companies decide if the data are appropriately de-identified?
    b. How and where are data stored?
    c. From your perspective, what are the benefits of these companies collecting the primary care data? What are the risks/downsides?

4. Why do other entities (like primary care clinics) share the primary care records with the for-profit companies?

5. Could you explain the processes by which for-profit companies get permission or consent to de-identify the data and create primary care datasets?
    a. Do patients consent to the de-identification and sharing of their data? Why or why not?
    b. What are your thoughts on this?

6. Do you think patients are aware their data are being used in these ways?
    a. How do you think patients or the general public would feel about their data being turned into primary care datasets owned by a for-profit company?

© 2025 Spithoff S et al. *JAMA Network Open*.

7. Based on your experience and knowledge, could you explain what happens if a third party (like a pharmaceutical company or a government) is interested in using the de-identified data for analytics?
    a. Can you walk me through what happens when a third party expresses interest?
    Prompt: Do for-profit companies share or sell the raw data to users or do they internally run the analytics and provide users with insights from a data set, or both?
    Prompt: what are the decision-making processes?

8. Documents produced by the for-profit companies that collect primary care data indicate that primary care datasets contain physician identifiers (eg names, addresses)
    a. Why would companies leave physician identifiers in the datasets?

9. What types of organizations or companies use the proprietary primary care datasets held or owned by for-profit companies?
    a. How do they use them?
    b. What benefit do they get from the datasets?
    c. From your perspective, what are the benefits to patients? Downsides? Marginalized groups?

10. (If not described already) How do pharmaceutical companies use the primary care datasets held/owned by for-profit companies?
    a. Without disclosing confidential information, can you provide examples?
    b. How is this data helpful for them?
    c. From your perspective, what are the benefits to patients? Downsides? Marginalized groups?

11. (If not described already) How do those in the **public sector,** such as governments, publicly funded researchers (or other entities mentioned by interviewee) use the proprietary datasets owned/held by for-profit companies?
    a. Can you provide examples?
    b. How is this data helpful for them?
    c. From your perspective, what are the benefits to patients? Downsides? Marginalized groups?

12. How do you feel, as a patient, about your data being turned into proprietary primary care datasets owned or held by a for-profit company?
    a. What do you see as the benefits? What do you see as the harms?

13. From your knowledge and experience, what considerations do for-profit companies that create and own datasets of de-identified patient records take for collecting, de-identifying and sharing/selling data from Indigenous Peoples?
    Prompt: How does a patient's status of Indigeneity affect how for-profit data companies get consent and permission for their data use?
    i. Is there a way for primary care data to be used to identify Indigenous patients?

14. What are the benefits of separating Indigenous People's data from the general population?
    a. What are the risks of doing so?

15. How do you think risks from the creation and use of primary care datasets owned/held by for-profit companies can be addressed?
    Prompt: could regulation address the risks? If so, how?

16. Is there anything else you would like to tell me?

© 2025 Spithoff S et al. *JAMA Network Open*.

eAppendix 2. Concept, Category and Code Descriptions

Concept	Category	Codes
Overview: Two main business models for commercialization data from primary care medical records in Canada	The conventional model	Descriptions of how the data brokers acquire, de-identify data, and monetize data from investor-owned chains of primary care clinics; descriptions of data broker clients and client relationships within this model
	The vertically-integrated model	Descriptions of how data brokers acquire, de-identify data, and monetize data from data broker-owned chains of primary care clinics; descriptions of data broker clients and client relationships within this model
Complex reciprocal relationships	Investor-owned chains of primary care clinics contribute and benefit	Explanations of why these chains are providing data to commercial data brokers; explanations of why commercial data brokers want data from chains of clinics
	Data brokers contribute and benefit	Explanations of how data brokers contribute by collecting, organizing and preparing data as well as by running analytics and interventions for pharmaceutical companies, academics and governments
	Pharmaceutical companies contribute and benefit	Explanations of how the pharmaceutical industry benefits from the data, data analytics and the targeting of patients with drug interventions
	Physicians contribute and benefit	Explanations of how physicians contribute by providing consent for data brokers to access to primary care data and to target patients with drug interventions; explanations about how physicians benefit
Tied to the interests of the pharmaceutical industry	The pharmaceutical industry is the data brokers' primary client	Explanations of how the pharmaceutical industry is the primary client of primary care data in the horizontal model; and the primary client/sponsor of data brokers (to identify and target patients) in the vertical model
	The corporate interests are made explicit in day-to-day activities of physicians and clinic management	Descriptions of physicians' experiences, as contractors at investor-owned chains of primary care clinics; their experiences receiving requests to consent to allow data brokers to access to patient records; and to participate in corporate initiatives and prescribe specific drugs; descriptions of role of clinical staff in ensuring physicians provided consent to allow data broker to access patient records and physicians prescribed specific drugs
	Benefits outweigh the risks	Improved access to new treatments increasing access to pharmaceutical treatments; Descriptions or perceptions that pharmaceutical influence over care is not harmful and may even be beneficial
Patients and Indigenous communities: little say in data collection and use	Patients' role in discussions about how their data are used	Explanation of the role of patients in consenting for data brokers to access, analyze and monetize patient medical records
	The social licence to collect and monetize patient medical records	Explanations of whether/how commercial data brokers have public support to gather and use primary care data; perceptions related to privacy risks and social licence; descriptions of social licence and concerns related to privacy and evaluations of their magnitude and severity
	Indigenous communities' role in discussion about how their data are used	Discussions and perspectives related to Indigenous communities regarding whether and how their data are used

© 2025 Spithoff S et al. *JAMA Network Open*.
