# Supplementary material for: The Primary Care Medical Record Industry in Canada and Its Data Collection and Commercialization Practices
Source: JAMA Netw Open. 2025 May 5;8(5):e257688. doi: 10.1001/jamanetworkopen.2025.7688 (PMC12053517; doi:10.1001/jamanetworkopen.2025.7688)
Supplement: Supplement 2. — Data Sharing Statement [file jamanetwopen-e257688-s002.pdf]

Data Sharing Statement

Spithoff. The Primary Care Medical Record Industry in Canada and Its Data Collection and Commercialization Practices. *JAMA Netw Open*. Published April 28, 2025. doi:10.1001/jamanetworkopen.2025.7688

Data
Data available: No

Additional Information
Explanation for why data not available: Data are available on reasonable request. The study interview guide is available as online supplemental file. Interview transcripts are not available to protect participant privacy.
